# Epigenetic Regulation of miR-25 and Lnc107153 on Expression of Seasonal Estrus Key Gene *CHGA* in Sheep

**DOI:** 10.3390/biology12020250

**Published:** 2023-02-04

**Authors:** Ran Di, Yekai Fan, Xiaoyun He, Qiuyue Liu, Xiangyu Wang, Yiming Gong, Joram Mwashigadi Mwacharo, Caihong Wei, Yufang Liu, Mingxing Chu

**Affiliations:** 1 Key Laboratory of Animal Genetics, Breeding and Reproduction of Ministry of Agriculture and Rural Affairs, Institute of Animal Science, Chinese Academy of Agricultural Sciences, Beijing 100193, China; 2 Institute of Genetics and Developmental Biology, Chinese Academy of Sciences, Beijing 100101, China; 3 Small Ruminant Genomics, International Center for Agricultural Research in the Dry Areas (ICARDA), Addis Ababa P.O. Box 5689, Ethiopia; 4 Animal and Veterinary Sciences, SRUC and Centre for Tropical Livestock Genetics and Health (CTLGH), The Roslin Institute Building, Midlothian EH25 9RG, UK

**Keywords:** sheep, seasonal reproduction, CHGA, miRNA, LncRNA, CeRNA

## Abstract

**Simple Summary:**

In sheep pituitary pars tuberalis tissue, *EYA3*, *TSHβ* and *CHGA* are key genes regulating seasonal reproduction. Their high expression is the characteristic signal under long photoperiods (*EYA3*, *TSHβ*) and short photoperiods (*CHGA*). The epigenetic regulation of microRNAs and LncRNAs on their expression remains unknown. In this study, our results indicated that miR-25 could inhibit the expression of *CHGA* by specifically binding to its 3′UTR region in pituitary cells. Lnc107153 played the role of CeRNA, which can adsorb miR-25 and weaken the inhibitory effect of miR-25 on *CHGA* expression. Together, these results suggested the epigenetic regulative network of Lnc107153-miR-25-*CHGA* was involved in sheep seasonal reproduction.

**Abstract:**

Pituitary pars tuberalis (PT) plays an important role as the transmission center in the seasonal reproduction of animals. It helps convert external photoperiod signals into intrinsic seasonal reproduction signals. In sheep PT, specific expression patterns of several genes (including short photoperiod-induced gene *CHGA* and long photoperiod genes *EYA3* and *TSHβ*) under different photoperiods are crucial characteristics during this signal transduction. Recent studies have revealed the role of epigenetics in regulating the expression of seasonal reproductive key genes. Therefore, we explored whether microRNAs and LncRNAs regulated the expressions of the above key genes. Firstly, the expression of miR-25 and *CHGA* showed a significant negative correlation in sheep PT. Results of the dual luciferase reporter assay and miR-25 overexpression indicated that miR-25 could inhibit the expression of *CHGA* by specifically binding to its 3′UTR region in pituitary cells. Then, expression negative correlation and dual luciferase reporter analyses were used to screen and identify the candidate LncRNA (Lnc107153) targeted by miR-25. Finally, the results of fluorescence in situ hybridization and Lnc107153 overexpression suggested that Lnc107153 and miR-25 were involved in the epigenetic regulation of *CHGA* expression. However, the expressions of *EYA3* and *TSHβ* were not regulated by miRNAs. These results will provide new insights into the epigenetic regulatory network of key genes in sheep seasonal reproduction.

## 1. Introduction

For many animal species, seasonal reproduction is the result of natural selection for increasing the survival rate of their offspring, thus their offspring can be born during the most appropriate stage of the annual climate and food supply [1]. However, for the sheep industry, seasonal reproduction has become an important factor limiting production efficiency [2,3]. The global demand for mutton is gradually increasing, and the current reproduction efficiency of sheep needs to be greatly improved [3]. Therefore, research on the molecular basis of the seasonal reproduction of sheep is crucial for possible artificial regulation of this trait in the future. Furthermore, the molecular regulatory network of seasonal reproduction remains to be improved in depth.

Pituitary pars tuberalis (PT) plays an important role as the transmission center in the seasonal reproduction of animals. It helps convert external photoperiod signals into intrinsic seasonal reproduction signals by the MEL-TSH-TH axis [4,5,6,7,8], which regulates the seasonal estrus of animals. In sheep PT tissue, specific expression patterns of several genes under different photoperiods are crucial characteristics during this signal transduction. For example, a short photoperiod (SP) induced a high expression of the *CHGA* gene [9], and a long photoperiod (LP) initiated the high expression of *EYA3* and *TSHβ* [5,6,7,8].

Recent studies have revealed the role of epigenetics in regulating the expression of seasonal reproductive key genes [10,11,12,13,14,15]. For example, it was reported that a short photoperiod inhibited hypothalamic DNA methyltransferase expression and reduced *dio3* promoter DNA methylation, which up-regulated *dio3* expression and induced gonadal regression [13]. In addition to DNA methylation, previous studies implied that microRNAs and LncRNAs were also important epigenetic regulators for seasonal reproduction [14,16,17,18,19]. It is worth exploring whether the specific expression patterns of the above key genes (*CHGA*, *EYA3* and *TSHβ*) are regulated by miRNAs and LncRNAs, which will expand our understanding of the epigenetic regulation of the key genes in sheep seasonal reproduction.

Sunite sheep is one typical seasonal-reproductive breed in Inner Mongolia, China, which rest from April to July every year [14]. Therefore, in this study, Sunite sheep were used and experienced the process from SP to LP after removing the ovaries and embedding estrogen. First, miRNAs targeting the above three key genes (*EYA3*, *TSHβ* and *CHGA*) were predicted with a sequence complementary relationship, and miRNA–gene target combinations with negative correlation in expression were screened using quantitative PCR results of miRNAs and genes under SP and LP in sheep PT. Then, the target binding relationship between miRNA and gene was verified with the dual luciferase reporter system, and the regulatory effect of miRNA on gene expression was analyzed using an overexpression experiment of miRNA in pituitary cells. Next, expression correlation and dual luciferase reporter analysis were used to screen and identify the candidate LncRNA targeted by miR-25. Finally, the regulatory network of LncRNA-miRNA-gene was revealed using expression correlation analysis, the dual luciferase reporter system and an intracellular LncRNA overexpression experiment. These results will provide new insights into the epigenetic regulatory network of the key genes in sheep seasonal reproduction.

## 2. Materials and Methods

### 2.1. Gene Expression Assay

Determination of gene expression in tissues was conducted in six adult Sunite ewes (3 years old; weight 40–45 kg). They were collected from a farm in Urat Middle Banner (40°75′ north latitude), Inner Mongolia Autonomous Region, China. All ewes were raised under the same conditions, with free access to water and feed. The surgery of ovariectomized (OVX), estradiol-implantation and the light control experiment referred to the methods previously reported [8,19,20,21,22]. Estradiol (E2, Sigma Chemical Co., St. Louis, MO, USA) was implanted to maintain plasma estradiol levels of 3–5 pg/mL [8,14,17,19]. After the surgery of OVX and estradiol-implantation, the ewes recovered for 30 days before artificial light control. Then, all the ewes were brought indoors with a light program simulating the outdoor photoperiodic condition using a time-control switch. Firstly, all ewes were kept in artificial SP (8 h light: 16 h dark) for 21 days and switched to LP (16 h light: 8 h dark) for 42 days, with free access to water and food. Ewes were euthanized (intravenous pentobarbital: 100 mg/kg) at ZT4 (4 h after lights on) of SP21 and LP42 [14]. Consistent with the specific location described by Wood et al. [9] and Lomet et al. [8], the PT tissue (the specific location is shown in Figure S1) of each ewe was immediately collected and stored at −80 °C.

Total RNA was extracted from PT tissues with TRIzol Reagent (Invitrogen, Carlsbad, CA, USA) according to the manufacturer’s instructions. The PrimeScript^TM^ reagent RT kit (Takara-Bio, Dalian, China) was used for genomic DNA removal and reverse transcription to synthesize the first strand of cDNA. After searching *EYA3*, *TSH*, *CHGA* and *RPL19* genes and Lnc20518, Lnc107153 and Lnc72519 sequences in our previous transcriptome sequencing results [14], the qPCR primers for the above four genes and three LncRNAs were designed using Premier 5 software (Table 1). *RPL19* was used as an internal reference to normalize target gene expression. The qPCR was performed using the SYBR premix ExTaq II kit (Takara-Bio, Dalian, China). The 20 μL qPCR reaction system included 1.0 μL cDNA template, 10 μL SYBR Premix Ex *Taq*, 0.5 μL forward and reverse primers and 8 μL ddH_2_O. The qPCR reaction program was as follows: 95 °C for 15 min, followed by 40 cycles of 95 °C for 10 s and 60 °C for 30 s. The expression levels were then calculated using the 2^−ΔΔCt^ method [23,24] and processed using SPSS 20.0 with a one-way analysis of variance.

The miRNAs which target *CHGA*, *TSHB* and *EYA3* genes were predicted using the miRBase database [25]. The first strand of miRNA cDNA was synthesized using the miRcute enhanced miRNA cDNA first strand synthesis kit (Tiangen biotech, Beijing, China) with total RNA as a template. MiRprimer2 was used to design the upstream primer sequences for miR-25, miR-30d and miR-22-3p (Table 2). Downstream primer was provided by the kit. The 20 μL reaction system included 2.0 μL miRNA cDNA template, 10 μL 2 × miRcute Plus miRNA premix, 0.4 μL forward and reverse primers and 7.2 μL ddH_2_O. The procedures for miRNA qPCR are shown in Table S1. The correlation of expression between genes (or LncRNAs) and miRNAs was analyzed using SPSS 20.0, including *EYA3*-miR-22-3p, *TSH*-miR-30d, *CHGA*-miR-25, Lnc20518-miR-25-*CHGA*, Lnc107153-miR-25-*CHGA* and Lnc72519-miR-25-*CHGA*.

### 2.2. Identification of the Targeting Relationship between miR-25 and CHGA

To verify whether there is a targeted binding relationship between miR-25 and *CHGA*, the *CHGA* 3′UTR sequence (Table S2) was inserted downstream of luc2 in the pmir-Glo vector and named as *CHGA* 3′UTR-WT. In order to ensure the accuracy of the test results, the binding site of miR-25 in *CHGA* 3′UTR was mutated, and the mutated *CHGA* 3′UTR was inserted downstream of luc2 in the pmir-Glo vector, which was named *CHGA* 3′UTR-MUT. The miR-25 mature sequence (AUUGCACUUGUCUCGUCUGA) was provided to Guangzhou Ruibo Biotechnology Co., Ltd. for miR-25 mimics synthesis. The micrON mimic NC with a base length of 21 nt was selected as the control. One of the two pmir-Glo vectors and one of the two mimics (Specific combinations are shown in the following experimental groups) were co-transfected into HEK 293T cells line using Lipofectamine 2000. Specifically, there were four experimental groups: ① *CHGA* 3′UTR-WT + miR-25 mimics; ② *CHGA* 3′UTR-MUT + miR-25 mimics; ③ *CHGA* 3′UTR-WT + mimics NC; and ④ *CHGA* 3′UTR-MUT + mimics NC. The luciferase activity of luc2/hRluc in transfected cells would be significantly reduced when the miRNA targeted the 3′UTR of the candidate gene.

RC-4B/C cells (rat adenohypophysoma cells) were purchased from American Type Culture Collection (San Diego, CA, USA). Cells were cultured in strict accordance with the instructions. Cells were cultured in 6-well plates with the same number of cells (10^6^) per well. Three biological replicates were set for each experimental group. When the cell density reached 80%, the experimental group was transfected with miR-25 mimics and the control group was transfected with mimic NC and the medium was replaced with OPTI-MEM. After 6 h, OPTI-MEM was replaced with a complete medium. After 48 h, cells were collected for qPCR and Western blot. The primers and detection methods of qPCR were the same as described above. Total cellular protein was extracted for concentration determination and Western blot. Anti-Chromogranin A Antibody (ab283265) was purchased from Abcam Company. GAPDH Mouse Monoclonal Antibody was purchased from Proteintech Company. HRP-conjugated Goat Anti-Mouse IgG (H+L) was purchased from Proteintech Company. The results of the Western blot were analyzed with Image J software. The difference significance test for the ratio of the target protein to GAPDH was performed using the chi-square test in SPSS 20.0.

### 2.3. Screening and Identification of LncRNA Targeted by miR-25

Another Sunite ewe was euthanized at ZT4 under the short photoperiod (SP21). PT tissue was immediately removed and fixed in 4 % paraformaldehyde (PFA). After washing, it was dehydrated and embedded with OTC. The tissue was cut into 10 μm sections using a freezing microtome and sections were pasted on slides. They were stored at −80 °C before fluorescence in situ hybridization. An Lnc107153 probe (sequence: 5′-cy3-AUAGAUCCUCCAGCUACCACCAUCAGACC-3′) labeled with CY3 was designed and synthesized by Bioengineering (Shanghai, China) Co., Ltd. The cell nuclei were stained with DAPI. When the fluorescence in situ hybridization ended, the images were collected using an upright fluorescence microscope.

The 3′UTR sequence (Table S2) of Lnc107153 was inserted downstream of luc2 in the pmir-Glo vector and named Lnc107153 3′UTR-WT. In order to ensure the accuracy of the test results, the binding site of miR-25 in the 3′UTR of Lnc107153 was mutated, and the mutated 3′UTR of Lnc107153 was inserted downstream of luc2 in the pmir-Glo vector, which was named Lnc107153 3′UTR-MUT. Four experimental groups were set up: Lnc107153-WT + miR-25 mimics; Lnc107153-WT + mimics NC; Lnc107153-MUT + miR-25 mimics; and Lnc107153-MUT + mimics NC. The pmir-Glo vector and mimics were co-transfected into the HEK293T cells line using Lipofectamine 2000. The luciferase activity of luc2/hRluc in transfected cells was detected.

In order to verify the role of Lnc107153 in pituitary cells, the Lnc107153 sequence (Table S3) was inserted into the vector pIRES2-EGFP to construct an overexpression plasmid (Lnc107153 + pIRES2-EGFP). Similar to miR-25 experiments, transfection was performed when the RC-4B/C cell culture density reached 80%. The experimental group was transfected with the Lnc107153 overexpression plasmid, and the control group was transfected with an overexpression empty vector. After 48 h, cells were collected, and total RNA and total protein were extracted for qPCR and Western blot, respectively.

## 3. Results

### 3.1. Prediction of the Targeting Relationship between the Key Genes and miRNA in Sheep PT

**Figure 1:**
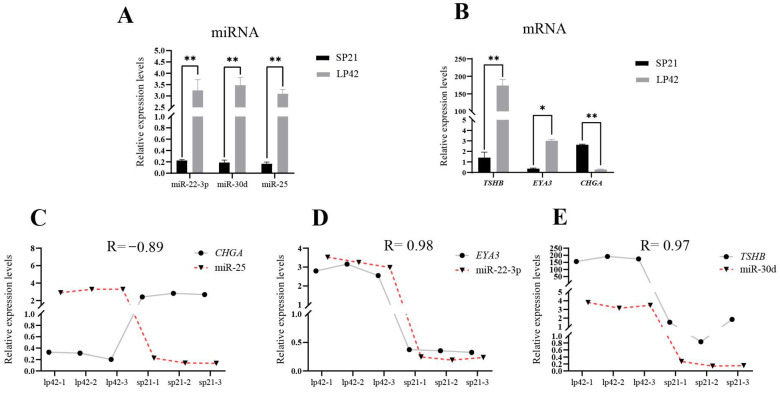
Expression level (**A**,**B**) and correlation analysis (**C**–**E**) of miRNAs and mRNAs in pars tuberalis of sheep under short and long photoperiods. * *p* < 0.05; ** *p* < 0.01.

In sheep PT tissue, *EYA3*, *TSHβ* and *CHGA* are key genes involved in the regulation of seasonal reproduction. Using information from the miRBase database, the top-ranking miRNAs targeting these three genes were predicted. They were miR-22-3p targeting *EYA3*, miR-30d targeting *TSHβ* and miR-25 targeting *CHGA*, respectively. The fluorescence quantitative PCR results showed that the three miRNAs had significantly higher expression levels during the long photoperiod compared to the short photoperiod in sheep PT (Figure 1A), and the expression of miR-25 was highly negatively correlated with the expression of *CHGA* (correlation coefficient: −0.89) (Figure 1A–C). Therefore, miR-25 was predicted to be a candidate miRNA negatively regulating the *CHGA* gene. However, for genes *EYA3* and *TSHβ*, there were no miRNAs that negatively regulated their expression (Figure 1D,E). Thus, the expressions of *EYA3* and *TSHβ* may be regulated by factors other than miRNAs.

### 3.2. Identification of the Targeting Relationship between miR-25 and CHGA

**Figure 2:**
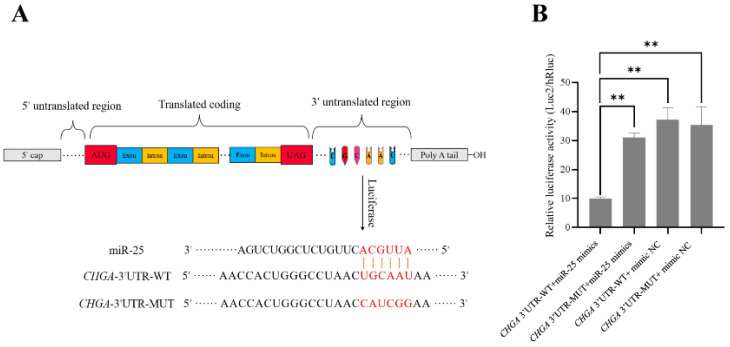
Complementary relationship between miR-25 and *CHGA* 3′UTR sequence (**A**) and results of dual luciferase reporter assay (**B**). ** *p* < 0.01.

**Figure 3:**
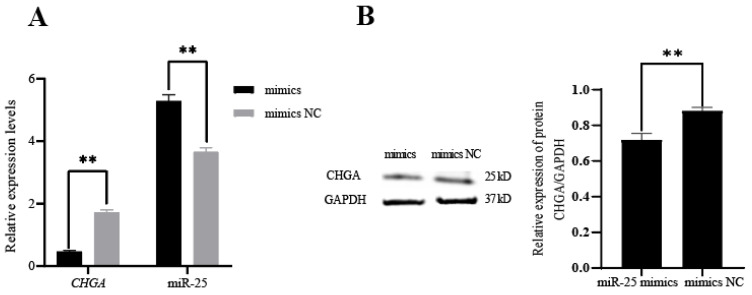
Expression of *CHGA* and miR-25 in RC-4B/C cells transfected with miR-25 mimics and mimic NC. (**A**) Transcriptional level; (**B**) translational level. ** *p* < 0.01.

Using the dual luciferase reporter system, we found that the relative fluorescence value of the co-transfected *CHGA* 3′UTR wild-type and miR-25 mimics group was significantly lower (*p* < 0.01) than those of the other three groups (*CHGA* 3′UTR mutant + miR-25 mimics; *CHGA* 3′UTR-WT + mimics NC; *CHGA* 3′UTR-MUT + mimics NC) (Figure 2), and there was no significant difference among the other three groups (*p* > 0.05). This result indicated that miR-25 can specifically bind to the 3′UTR region of *CHGA* through base complementation. Furthermore, the miR-25 mimics and mimic NC were transfected into pituitary cells (RC-4B/C cells) that can express *CHGA*, respectively. The qPCR results showed that the expression of miR-25 was significantly higher in the miR-25 mimics overexpression group than that in the mimic NC group (*p* < 0.01), which indicated that miR-25 was successfully transfected. After transfection of the miR-25 mimics, the transcription and translation levels of *CHGA* were significantly decreased (*p* < 0.01) (Figure 3A,B). In summary, miR-25 can inhibit the expression of *CHGA* in pituitary cells by specifically binding to the 3′UTR region of *CHGA*.

### 3.3. Screening and Identification of LncRNA Targeted by miR-25

**Figure 4:**
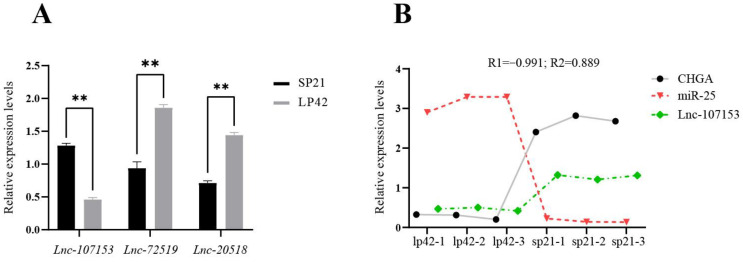
Expression of three LncRNAs in sheep PT (**A**) and correlation analysis of *CHGA*-miR-25-Lnc107153 expression under short and long photoperiods (**B**). R1 represents the correlation coefficient between Lnc107153 and miR-25; R2 represents the correlation coefficient between Lnc107153 and *CHGA*. ** *p* < 0.01.

Based on our previous transcriptome sequencing data [14], we predicted that targeted complementary relationships existed between miR-25 and three LncRNAs (Lnc107153, Lnc20518 and Lnc72519). Next, the results of the qPCR in sheep PT under different photoperiods showed that the expression of Lnc107153 during the short photoperiod was significantly higher than that during the long photoperiod (*p* < 0.01), and the expression of Lnc107153 was highly negatively correlated with the expression of miR-25 (Figure 4, correlation coefficient: −0.991). For the expression of the other two LncRNAs (Lnc20518 and Lnc72519), there was no negative correlation found with miR-25 (Table S4). Notably, a high positive correlation of expression existed between Lnc107153 and *CHGA* (Figure 4B, the correlation coefficient is 0.889). Therefore, the regulative network Lnc107153-miR-25-*CHGA* was screened for subsequent validation.

**Figure 5:**
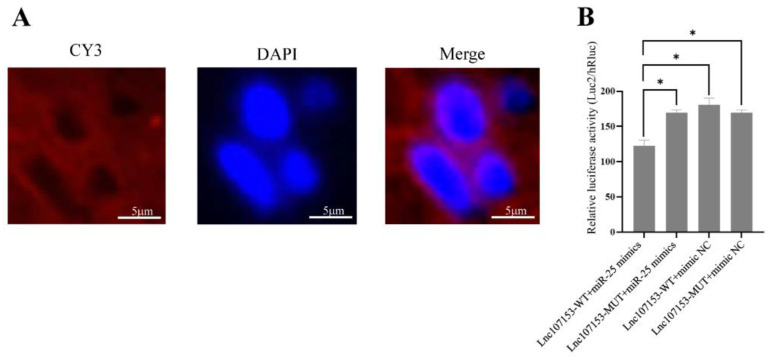
Fluorescence in situ hybridization results of Lnc107153 in sheep PT (**A**) and dual luciferase activity detection of miR-25 binding to Lnc107153 3′UTR (**B**). Lnc107153 was labeled with CY3 (red fluorescence); the nucleus was labeled with DAPI (blue fluorescence). * *p* < 0.05.

Firstly, Lnc107153 was localized in the cytoplasm of sheep PT cells with fluorescence in situ hybridization (Figure 5A, Lnc107153 with CY3 red marker), suggesting that Lnc107153 has the potential to competitively bind miRNA as a CeRNA. Next, the results of the dual luciferase reporter assay (Figure 5B) showed that the relative fluorescence value of the transfected Lnc107153-WT + miR-25 mimics group was significantly lower than those of the other three groups (Lnc107153-WT + mimics NC, Lnc107153-MUT + miR-25 mimics, Lnc107153-MUT + mimics NC), indicating that miR-25 can specifically target the 3′UTR region of Lnc107153.

**Figure 6:**
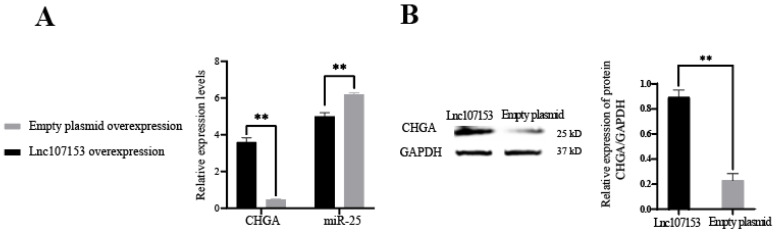
Expression levels of *CHGA* and mir-25 in RC-4B/C cells after overexpression of Lnc107153 and empty plasmid vector. (**A**) Transcriptional level of *CHGA* and mir-25; (**B**) translational level of CHGA. ** *p* < 0.01.

**Figure 7:**
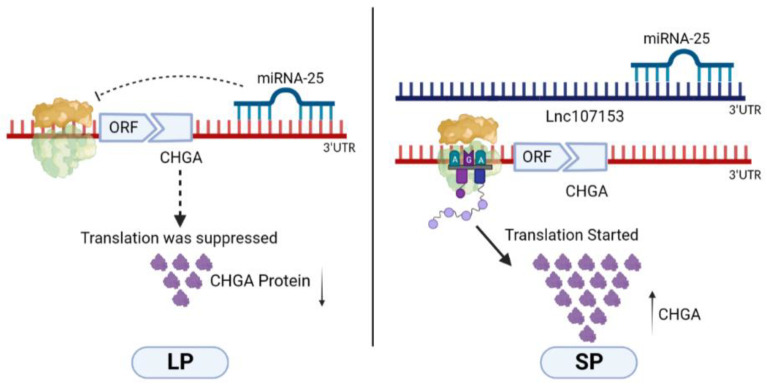
Epigenetic regulative network of Lnc107153-miR-25-*CHGA* involved in sheep seasonal reproduction.

In order to further verify the CeRNA function of Lnc107153, the Lnc107153 overexpression plasmid and the control empty overexpression plasmid were transfected into RC-4B/C cells, respectively. Together, the results of the qPCR and Western blot showed that the overexpression of Lnc107153 significantly reduced the level of miR-25 and increased the transcription and translation of the *CHGA* gene (Figure 6). Combined with the above result of this study that miR-25 can inhibit the expression of the *CHGA* gene, it can be speculated that Lnc107153 might play the role of CeRNA to adsorb miR-25 and weaken the inhibitory effect of miR-25 on *CHGA* expression in pituitary cells. Therefore, the regulative network Lnc107153-miR-25-*CHGA* might be summarized as (Figure 7): during the long photoperiod, miR-25 was highly expressed in sheep PT which could specifically bind to the 3′UTR region of *CHGA* to inhibit CHGA protein expression; during the short photoperiod, the expression of Lnc107153 increased, which could bind to miR-25 to weaken the inhibitory effect of miR-25 on *CHGA* expression, and consequently, the expression level of CHGA protein increased significantly.

## 4. Discussion

In sheep, the retina receives light signals of different seasons and day lengths, then the signals are transmitted to the pineal gland via suprachiasmatic nuclei (SCN) [26]. In the pineal gland, the continuous secretion duration of melatonin (MEL) changes accordingly [27,28,29]. After MEL receptors in the pituitary pars tuberalis perceive the dynamic changes in MEL, the expression levels of a series of photoperiod-induced genes (such as the SP-induced gene *CHGA* [9], LP factor *EYA3* [6], *TSHβ* [30] and *BMAL2* [31]) will undergo a landmark alteration. These photoperiodic-induced genes can help convert photoperiodic signals into intrinsic seasonal reproduction signals by the MEL-TSH-TH axis, then they directly or indirectly affect the release of gonadotropin-releasing hormone (GnRH) in the hypothalamus and further regulate the reproductive phenotype through the hypothalamic–pituitary–gonadal axis [4,32].

In this study, the results of the qPCR in sheep PT showed that the expression of *EYA3* and *TSH* under the long photoperiod was significantly higher than that of the short photoperiod, and the expression of *CHGA* exhibited the opposite trend. These results were consistent with previous reports [5,6,9] and proved the reliability of transcriptome sequencing results. *EYA3* is involved in the regulation of the seasonal reproduction of animals as a long photoperiod signal. A study on quail revealed that EYA3 is the first gene to be activated and induced to express in PT on the first day of the long photoperiod [33]. Studies on sheep have shown that the LP initiates the high expression of *EYA3* [6] and further activates *TSHβ* transcription mainly through the TEF/SIX1/EYA3 complex [34]. Wood et al. [9] revealed that *CHGA* was involved in the regulation of the seasonal reproduction of sheep as an SP signal using RNA-seq analysis and fluorescence immunoassay. Their results showed that the transcription level of *CHGA* under SP was three times that under the LP in sheep PT, and the level of CHGA protein was also significantly higher than that in the LP. High levels of CHGA could make thyroid-stimulating cells surrounded by stellate follicular cells [9], resulting in a decrease in T3 concentration in the hypothalamus. A low concentration of T3 relieved the encirclement of ependymal cells on GnRH neuronal terminals, thus the release of GnRH increased [6,35], which would promote animal estrus. CHGA is a signal factor secreted by chromaffin granules of neurosecretory cells. Its biological functions in cells are mainly concentrated in two aspects: one is to selectively regulate the accumulation of target peptide hormones and neurotransmitters on the Golgi network membrane, and the other is that the CHGA protein can be hydrolyzed into angiostatin-I, angiostatin-II and catechins to promote the permeability of the Golgi network and participate in Ca^2+^ and catecholamine metabolism [36].

MicroRNA can bind to the 3′UTR region of the target gene to inhibit protein translation. It has been previously reported that other miRNAs inhibited *CHGA* expression by binding to its 3′UTR. For example, in the study of the *CHGA* 3′UTR variant C + 87T (rs7610), Zhang et al. [37] found that miR-107 has a sequence complementary to *CHGA* 3′UTR and the expression level of *CHGA* in human neuroblastoma cells is opposite to the expression level of miR-107, and then verified that miR-107 can inhibit *CHGA* expression by targeting its 3′UTR using double luciferase reporter system. When Fu et al. [38] screened differentially expressed genes and miRNAs in gastric cancer cells from the expression profile in the GEO database, they found that miR-125b-5p and miR-199a-5p had a targeting relationship with *CHGA*. These two miRNAs were also found in our sequencing results, but their expression levels did not show significant differences between different photoperiods (not shown), suggesting that miRNAs function in a tissue-specific manner. Current research on miR-25 focuses on diseases of fat, blood vessels and the pancreas [39,40], but less on brain tissue and reproductive tissues. In this study, the targeted relationship between miR-25 and *CHGA* was verified with a dual luciferase report, overexpression, qPCR and Western blot experiments, which provided new insights into the regulatory role of miR-25 in the animal pituitary.

LncRNA is a kind of long non-coding RNA with a similar base structure as mRNA. This feature enables LncRNA as a sponge to bind a variety of miRNAs, thereby relieving the inhibition of miRNAs on their target genes and increasing the expression level of target genes. This effect is also known as the competitive endogenous RNA (CeRNA) mechanism [41,42], which exists in the cytoplasm [43,44]. In sheep, there were also reports on the function of LncRNA as CeRNA. For example, Yao et al. [45] found that LncRNA FDNCR as a molecular sponge can absorb miR-543-3p in granulosa cells of Hu sheep, thus it prevented miR-543-3p from binding to 3′UTR of the target gene DCN, consequently activated DCN and finally promoted granulosa cell apoptosis. Combining the results of fluorescence in situ hybridization, the dual luciferase reporter assay, overexpression, qPCR and the Western blot, our study revealed that Lnc107153 could act as a CeRNA to regulate the expression of CHGA by competitively binding to miR-25. This result will help us further understand the epigenetic regulation mechanism of photoperiod-induced key genes in sheep seasonal reproduction. In addition, these results suggest that it is possible in the future that, under long photoperiods, Lnc107153 can be used to increase the expression of CHGA, and then the estrous season of sheep will be extended through the TSH-TH axis. In this way, the results also suggest that microRNAs and LncRNAs are involved in the regulation of seasonal estrus in sheep. The molecular regulatory network of animal seasonal reproduction can be explored from the perspective of epigenetics.

## 5. Conclusions

In sheep PT tissue, *EYA3*, *TSHβ* and *CHGA* are key genes regulating seasonal reproduction. The epigenetic regulation of microRNAs and LncRNAs on their expression remains unknown. In this study, our results indicated that miR-25 could inhibit the expression of *CHGA* by specifically binding to its 3′UTR region in pituitary cells. Lnc107153 played the role of CeRNA, which can adsorb miR-25 and weaken the inhibitory effect of miR-25 on *CHGA* expression. Together, these results suggested the epigenetic regulative network of Lnc107153-miR-25-*CHGA* was involved in sheep seasonal reproduction.

## Figures and Tables

**Table 1:** Primer sequences for qPCR of four genes and three LncRNAs.

Genes/LncRNAs	Primer Sequences (5′-3′)	Length (bp)	Amplification Efficiency
*EYA3*	F: ACCCTTCTCCAAGCCCATC	232	97%
	R: ACTGTTGGGTCCTTTCCATACTT		
*TSHβ*	F: CCTAACCATCAACACCACCAT	211	98%
	R: ATTACACTTGCCACACTTACAGC		
*CHGA*	F: GAATAAAGGGGACACTGAGGTGAT	112	96%
	R: TCCTCGGAGCGTCTCAAAAC		
*RPL19*	F: TCTAAGAAGATTGACCGCCACAT	240	99%
	R: CTCCTTGGACAGAGTCTTGATGATT		
Lnc107153	F: AATATGGGTCTGATGGTGGTAGC	187	98%
	R: TGCCTTTGCCATATAACGTAGC		
Lnc72519	F: ATGAAGTGATGGGACCAGATG	149	97%
	R: GATGAAGCACAAGCTGGAATC		
Lnc20518	F: CCACAAGGGAAGCCCATAGT	244	97%
	R: AGGTGACTTCCAAATCTTGGCT		

**Table 2:** Mature sequences and forward primer sequences for qPCR of three miRNAs.

miRNAs	Mature Sequences (5′-3′)	Forward Primer Sequences (5′-3″)
miR-25	AUUGCACUUGUCUCGGUCUGA	F: GATTGCACTTGTCTCGGT
miR-30d	UGUAAACAUCCCCGACUGG	F: GCAGTGTAAACATCCCCGA
miR-22-3p	AAGCUGCCAGUUGAAGAACUG	F: GAAGCTGCCAGTTGAAGA
u6	-	F: CTCGCTTCGGCAGCACA

## Data Availability

All the data in this study were shown in figures, tables and supplementary material files.

## Supplementary Materials

The following supporting information can be downloaded at: https://www.mdpi.com/article/10.3390/biology12020250/s1. Figure S1: The location where the sheep pituitary pars tuberalis was collected in this study; Table S1: qPCR procedure for miRNAs in this study; Table S2: Sequence information of wild-type and mutant-type 3′UTR of *CHGA* and Lnc107153; Table S3: Sequence information of Lnc107153 in sheep PT; Table S4: Pearson correlation coefficient of expression pattern of LncRNA-miRNA-mRNA.
