# Supplementary material for: Epigenetic Regulation of miR-25 and Lnc107153 on Expression of Seasonal Estrus Key Gene CHGA in Sheep
Source: Biology (Basel). 2023 Feb 4;12(2):250. doi: 10.3390/biology12020250 (PMC9952879; doi:10.3390/biology12020250)
Supplement: Supplementary file 1 [file biology-12-00250-s001.zip › Table S1.pdf]

Table S1 qPCR procedure for miRNAs in this study

Program Name	Condition	Cycles
Pre-incubation	95℃ 15 min	1
Low abundance miRNA enrichment	94℃ 20 s<br>64℃ 30 s<br>72℃ 134s	5
Amplification	94℃ 20 s<br>64℃ 34 s	40
Melting curve	95℃ 5 s<br>65℃ 1 min<br>97℃ 5 s	1
Cooling	4℃30 s	-
