# Supplementary material for: Epigenetic Regulation of miR-25 and Lnc107153 on Expression of Seasonal Estrus Key Gene CHGA in Sheep
Source: Biology (Basel). 2023 Feb 4;12(2):250. doi: 10.3390/biology12020250 (PMC9952879; doi:10.3390/biology12020250)
Supplement: Supplementary file 1 [file biology-12-00250-s001.zip › Table S2.pdf]

Table S2 Sequence information of wild type and mutant type 3'UTR of *CHGA* and Lnc107153

Name of vectors	Sequence of the inserted 3'UTR
CHGA 3'UTR -WT	**GCTAGC**CCCTGCCCCAGTGCGTCCCGCTGCAGGGCAGACCCCTTGCCTTTCAACGATTATCCTGTCTCTGAACACAGGCAGCCTTCTCAAAGTTTCCTAGAACCACTGGGCCTAAC**TGCAAT**AAGTACTGACCTTTAGGTGAAAGCTGAGGACTCCTGACTATATTGTGTATGACATTTATCTAAGGGAAATAAATCTGCTCTGGGCTCTTTCCCTCGAG
CHGA 3'UTR -MUT	**GCTAGC**CCCTGCCCCAGTGCGTCCCGCTGCAGGGCAGACCCCTTGCCTTTCAACGATTATCCTGTCTCTGAACACAGGCAGCCTTCTCAAAGTTTCCTAGAACCACTGGGCCTAAC**CATCGG**AAGTACTGACCTTTAGGTGAAAGCTGAGGACTCCTGACTATATTGTGTATGACATTTATCTAAGGGAAATAAATCTGCTCTGGGCTCTTTCCCTCGAG
Lnc107153-WT	**GCTAGC**AGCCACTGACTGTGTGGTTTCTCTGTTATTCTAGTAGTTTAACCAATATGTAACTTTATTTATCATGCTGATTTCTCATTTCTAC**TGCAAT**CCAGTTCAGTTAGGGGTCTAAACAAGGAGCTCTGTTTCATGCAGTCATCTTGTGATCAAGGATTGGTCTCTCTGAAACCCAAAATATCATATAAGACAATGCTACTGAATGCCCTGGATCCAGTCGGTGAACTCGAG
Lnc107153-MUT	**GCTAGC**AGCCACTGACTGTGTGGTTTCTCTGTTATTCTAGTAGTTTAACCAATATGTAACTTTATTTATCATGCTGATTTCTCATTTCTAC**GATCGG**CCAGTTCAGTTAGGGGTCTAAACAAGGAGCTCTGTTTCATGCAGTCATCTTGTGATCAAGGATTGGTCTCTCTGAAACCCAAAATATCATATAAGACAATGCTACTGAATGCCCTGGATCCAGTCGGTGAACTCGAG

The 5’NheI was shown by thickening, the 3’XhoI was shown by underlining, and the mutated bases were shown by thickening underlining.
