# Supplementary material for: Epigenetic Regulation of miR-25 and Lnc107153 on Expression of Seasonal Estrus Key Gene CHGA in Sheep
Source: Biology (Basel). 2023 Feb 4;12(2):250. doi: 10.3390/biology12020250 (PMC9952879; doi:10.3390/biology12020250)
Supplement: Supplementary file 1 [file biology-12-00250-s001.zip › Table S3.pdf]

Table S3 Sequence information of Lnc107153 in sheep PT

LncRNA	Sequence
Lnc107153	TTATCCCCATTGTGTTCATGTTTCTCTTTAAATCTTTAAGT<br>ATTTCCATACCAGATTGTTTTACCTTTTTTGCTAATTCATT<br>TATGAAATTGTATGTGTCTGTTGACTAATACTTTTCTTGC<br>ATGTGAGTCAAATTTTCTCTCTTCTCATGTCTGTTGAAAC<br>ATTTTTAAATTGATTGATAAAAACCATTGAGAGAGATTTG<br>TTGGATACTTTTGAGAAGTATTGAATTTTGTTTTGACATT<br>AATTTACTGTAAATCAGTTTTGCATTTTAGAGGTTTTTTT<br>TTAACTTTATTTGAGTATTTATAGGTTAGAATTTATTTCAT<br>TATCTTTCTAAGATATCTTGTTTCTGGGGTCTATTAAGGTC<br>TCCAGTCTGGTTTTTCAGTTAGGTCTCTCCAGTCCACAAA<br>ACTTCTTTGTGTAGTTTTAGTCTATGCATTGGACCAAAGC<br>CCCCACCACCCCTATTCAGATTACTATAGCTCTTTCTCAA<br>CATCTTTCTCGACTTTTTGGTACTCTCTCCTACAAATTCCA<br>ACTGCCAAGCTTTTTTGAATTTCACAAGCTTTATCTTTAAT<br>TCATTGTGACCTTTCAATTCCTCTCTTCATCATCAATATCA<br>ACCAAATGCTTGATGGCTGAGGCATTAAATGTATGCTAA<br>GTCACGTCAGTCATATCTGACTCTGTGTGACTCTGTGAAC<br>TGTAGCCTCCCAGGCTCCACTGCCCATGTGATTCTCCAGG<br>CAAGAATACTGGAGTGGGTTGCCATGCCCTTCTCCAGGA<br>AACTTTCCAACCCAGGGATCGAACCCACATCTCTTTTGTG<br>TCCTGCATTGGCAGGTGGGTCCTTCACCACTAGCATCACC<br>TGGGAAGCCCAAGGCATTATATAGCTCATCCTTTTTTTTT<br>TCTTTTTGTCTTATTTCAGGGATTACAGTCATCTGCTGCTG<br>ATTTTCAGTATTTGATAACTGTTGTGTTATTTATTCTATTT<br>ATCTAAGGCAGAGGACTTAGCTCTTACTAAGTTTCATCAG<br>GATCTTCTATCTTATAAAAATCATTTTAAAGCATGAGAGA<br>TTGTATTAGTTAAAGGCAAGTTTCCCATGATCCTCATTTG<br>TAACAATTCTGGAAGTTAGTGGATATGATTAATGTGGGC<br>ATCATCAAGAAAATACCAAATAAATTTAGAAATTACAAG<br>TAATTCCAAGTAATTTCATCTTTATTCTCAGAGTTCAGTA<br>CTTAATTCTGCTAAAAACCATGGCAGGTACTCAGTAGTAT<br>GTAAAGAAAGGAGAATGACCCCCCAAAATGGTTTTTCTT<br>GGTATAATATTTCTCTTTCTATATCAACTACTCAAGGACA<br>AAAATCTTATATTTAATTGCTTAAAACCATAGATAAACAG<br>ATTAATTTTGTCACTATATGTGAGTTGCTCTCTACCAAAT<br>AACCCTCTTTAGTTTTCTGTAATAATAAAAGATCACTTTT<br>AACAAGCCTAAGGCAACCTACCAAAACCTATCTTTCCAC<br>TTCCCTTTGAAGCTAAATGTTTCCCTGTAACAAAGGTTTG<br>GCCAATAGAGTGTGAGGGTAACTAATGCATTTAATACCT<br>GAATTGTGATCATAAGAGAAAAGATATGTGCCTTCCCTTC

	CTTTTTTCCTGTTTCTTTCTCTCTCTCTTCTTCTTCTTCTTTT<br>TTTTTTTTTTTGAATATGGGTCTGATGGTGGTAGCTGGAG<br>GATCTATTTTTAATTCTGAGAAAGAAAGTTATGCTTTAAA<br>GTTAGCAGAATAATAAAAATAGAAGGTGCCTGAGGATGG<br>AGGGCTGAAAGTGGCCTCACAGAAGCTATGCACATCCTA<br>ATCCTTGGAACATGTGAATGCTACGTTATATGGCAAAGG<br>CAACCTGCATATATGATTAAGGTTCTCAAGATAAGGATA<br>TGATTCTGGTAGGCTCTAAGTGTAATTATCCGGGTCCCCA<br>AATAGGAGAAGCAAGAAAGTCAAAGAAGAAATTGGGAG<br>ATGTGATGACAAAAACAAGAAGTTGGAGTGATTTGAAGA<br>ATTAGTAATAGACCAAGGATTGTAGGTAACCCAAGAAGA<br>TAGAAATTCCAAGGATGTGGAGTTTCCTCTAGAGTCTCCA<br>AAATGAACCAACCCTGCTGGCACCTTAGTAAAACTTAGT<br>TTTGATTTCTGGCCTTCAGAAATGTAAGGAAATAAATTTA<br>GGTTGCTTAATATGTTACAGCAGGAATAAGAAACAAATG<br>CATTGGGTGTGCAATGAGAACAAGTTCCTCAATCATTATC<br>TGGCTGCCTACAAGTCTAATATCTTGTTTTAGACAAGGTA<br>TTTGGTTCTGTGTTAAAATAGCTTAATCCATAGCCATACT<br>ATGAAGCCACGATACCACTTTATGATGACATATCAGGAT<br>GTATATCTTACTTAAGCCACTGACTGTGTGGTTTCTCTGTT<br>ATTCTAGTAGTTTAACCAATATGTAACTTTATTTATCATG<br>CTGATTTCTCATTTCTACTGCAATCCAGTTCAGTTAGGGG<br>TCTAAACAAGGAGCTCTGTTTCATGCAGTCATCTTGTGAT<br>CAAGGATTGGTCTCTCTGAAACCCAAAATATCATATAAG<br>ACAATGCTACTGAATGCCCTGGATCCAGTCGGTGAACTG<br>TTTGTTACCTGGGCCTGGTTATGAGATAAAATACAGCAA<br>GTATGAGTAAGCTTCAGCATTTGTATAGTTAGTTCATATT<br>GCACCATGACCTATAAAAGAGATCTTATCTGAGTAACTG<br>TAAAAATTGAAGGGGAGGGGGTGGATATCTCCACACTGC<br>AAATAGAATCGTTTTCCTCAAGCACTGGAGAGACCACAT<br>GTGAAGGACTGTGTAGGAGATTTTTTAAGAGTAGGACTT<br>GTGGTATCAGATAGTGTTTTGCCTGCTTAGCAAGACACAT<br>AATTAGTGTGTTTGACATAAAGAAATCTGAGAAAAGTAA<br>AGGAGCTTTGGCTCAAAAAGAAGAAAGGATATATTTCAT<br>GTACATTAAGCCTGGTTTTGCCACAAGAGCCCAGTGTTCA<br>TAAACTTTCTGGGAGAAAGATCAGTCCATGCAGCCTGAT<br>AGCGCTCTCAGAAAGCAGATAAGACAATCATATTTCCGC<br>CAAGATGGAATTACTTGCATTTGTGAAAGTAGGTCCCTA<br>GAGATTACACCAAACCCCCATGCTGTTTTAGCTGGCATAT<br>ATCTTCTCCCTGATTCTGAAACAGAAAATGAGATTAACGT<br>TTCTACAGCCAGTGCTTTTGGGGCTGCTACAAAGCTAAAC<br>TTCCCGTGAACTCCACAATATTTCATACCCTCAGTCTGAA<br>AGTTTCTAGATCCTGTGCTCTATTAATTTCTGCTACATTTC<br>CTCTTGTTTCTAATACTGTCTTGCTCTTAACTGTTCCATTC

TTTCTCTCAGAAAGAATAAGCCACTTCATCTGGCTTTTTC
GTTTTGTAAGGCAGTGTGGCAGAAACAAGGGTCTTCCTTC
TCTTTTATTGCTCTCTGCTTTCTGGTCCACACATATTTGTT
CCCATGGATTTCTTATATGTAGTCTTATGGATTTGTCACA
GTGAGAGATTTGAAAATATTGCATCTAGATCTGCCAAAA
GCATGTATAGTTCAAGCAAAAGTTTTCCATGCTTTCCTTA
CTGATTTGAGTAATCCCACCCAGGGGCAGTTCTTCCATTA
GAATGATATCTAACCTACTCCCAGACATGATCCATCATTA
TCTTGCCTAGAGTCCTTTGGGTATGTTTCACCTTGGCTGT
GTTTTCACTCAGAGTCCTGATTCCAAACACTGCCAGATCA
TGAGTCCTTAGGCGCAGAGGACAACTTCATGACCACCTG
CAATAGCTGTCGCTTTAGAATACATCCCTGGATGCCAGG
GGAAAGTTTTACTGTCCATCTGTCTTTCTAATGTATTTTAC
ACAGTTTGTGCAAACACTTGATAGGAATTTATGGGCTGCT
ATTCTTACACTTGAGTAACCATAACTAAAATTATTATTCC
TATTTTTGTGTATATCTAACTTCACATTTATGAGTTAAGTC
GCACCCTTCCTCTGCTTAAAATTCTAAAATGACTTCTAAT
GTCACTCAAATTAAAAGCCAGTCTCTTGAAAGCCCATCA
AAACCATAAGTTTTCTGATTTTGTAGTATCTTCCTATCCTG
TTCGCCTCTCTCTTGCAAACTCCACTCTATCTGCTCTTAGT
GTCCACTGCTTTTCTTTTCTTTAGTATAACCAGTTTGCAGG
CATCTATTCCCTAAACCTGAAGATATTTAGCCAAATGCCA
ACAAGGCTCAGTTTCAACCACTTTAAACTGTTCAGTTAAA
TGTCCTTTTCTCAACAAAGACTTATCCAAATTATACAGTT
TAAGATTAGGACTAATCCCCATCTTCCCACAGTCTTGGTC
CCTCTTGATTTTATTTATGGATAGTGCTATTACTTTCTAAT
AAGATGTATATAGCCTAGAAAAGTATTAACATTATTTCCT
CATTATGTCAACATCTTTGAAATAGTGATACATTCTATAT
ATAAAAATGGGTATAAAAACAAAGTGTCTATCAACTAGC
AATGTTTCCTTCTTCGTTCAGTTCAGTTCAGTTCAGTTCAG
TCACTCAGTCGTGTCCTACTCTTTGCGACCCCATGAATCG
CAGCACGCCAGGACTCCCTGTCCATCAGCAACTCTTGGA
GTTCACTCAGACACACATCCATCAAGTCCATGATGCCATC
CAGCCATCTCATCCTCTGTCGTCCCCTTCTCCTCCTGCCCC
CAATCCCTCCCAGCATCAGAGTCTTTTCCAATGAGTCAAC
TCTTCACATGAGGTGGCCAAAGTACTGGAGTTTCAGCTTT
AGCATCATTCCTTCCAAAGACATCCCAGGGCTGATCTCCT
TCAGAATGGACTGGTTGGATCTCCTTGCAGTCCAAGGGA
CTTTCAAGAGTCTTCTCCAACACCACAGTTCAAAAGCATC
AATTCTTCGGCATTCAAACTTCTTCACAGAGACATAAATA
GTAGCATCTTAAATTTAATTAAATAAGGTCAATTTTACCT
AATAAGTTTATAGTTCAGTTTGGGCCTTC
