# Supplementary material for: Epigenetic Regulation of miR-25 and Lnc107153 on Expression of Seasonal Estrus Key Gene CHGA in Sheep
Source: Biology (Basel). 2023 Feb 4;12(2):250. doi: 10.3390/biology12020250 (PMC9952879; doi:10.3390/biology12020250)
Supplement: Supplementary file 1 [file biology-12-00250-s001.zip › Table S4.pdf]

Table S4 Pearson correlation coefficient of expression pattern of LncRNA-miRNA-mRNA

	CHGA	miR-25	Lnc20518	Lnc107153	Lnc72519
CHGA	1	-0.893*	-0.915*	0.889*	-0.789
miR-25	-0.893*	1	0.987**	-0.991**	0.974**
Lnc20518	-0.915*	0.987**	-	-	-
Lnc107153	0.889*	-0.991**	-	-	-
Lnc72519	-0.789	0.974**	-	-	-

*P<0.05， ** P<0.01.
